# Examination of the Dimensions of Biological Age

**DOI:** 10.3389/fgene.2019.00263

**Published:** 2019-03-26

**Authors:** S. Michal Jazwinski, Sangkyu Kim

**Affiliations:** Tulane Center for Aging, Department of Medicine, Tulane University Health Sciences Center, New Orleans, LA, United States

**Keywords:** deficit index, frailty index, biological age, complexity, network, gut microbiome, DNA methylation, healthy aging

## Abstract

The concept of biological age has been used more and more frequently in aging research in attempts to measure the progress of the biological aging process as opposed to the simple passage of time. Several approaches to quantify biological age have been utilized, including the use of biomarkers in the form of serum analytes, epigenetic markers, and deficit or frailty indices. Among these methods, the deficit index possesses a theoretical basis grounded in systems biology by incorporating networks, with their emergent properties, to describe the complex aging system. Application of the deficit index in human aging studies points to the increased energetic demands posed by an aging system that is losing integration. Different aspects of mitochondrial function appear to be responsible in males and females. The gut microbiome loses complexity in tandem with the host, as biological age increases, with likely impact on host metabolism and immunity. Specific DNA methylation changes are associated with biological age. They suggest declining connectivity within the aging network, at the cellular level. The deficit/frailty index may account for at least part of the departure at older ages of the observed mortality in the population from the exponential increase modeled by the Gompertz equation.

## Introduction

Even to the untrained eye it has always been apparent that different people age differently. What this really means is that the perceived age may vary from the actual chronological age, based on the usual presentation of individuals at a given calendar age in any given time and place. Subjective evaluation of age rather accurately assesses the ravages of time and coincides quite adequately to more objective metrics (Christensen et al., 2009). Nevertheless, we would like to be able to reference such objective measures to examine in greater detail the dimensions of aging.

The dimensions of aging encompass at least three different aspects. The first incorporates prediction of survival or mortality. In other words, we want to be able to relate a process, aging, to an outcome, longevity. This has long been a domain of aging research, and it continues to engage biodemographers. The second attempts to relate an aging process to the ability to function. So-called healthy aging derives from this approach. Finally, the need to evaluate potential therapies or interventions to extend this healthspan is yet another dimension.

There are two facets of this discussion that have not been explicitly addressed thus far. One is the accommodation of change that occurs with time. Any measure of aging must be able to allow us to incorporate this dynamic feature of the aging process. Indeed, the capacity to treat aging as a process requires this. Secondly, we must acknowledge that aging occurs at different levels of inquiry, biological, psychological, sociological, etc. All these levels address functioning, but from different vantage points. Thus, the direction we take should be applicable from these several points of observation.

Our focus in this article is biological or physiological. However, the direction we take can be readily utilized in other disciplinary venues. An illustrative example in this regard is the use of two terms that refer to the measure of functional aging we explore in detail below. Originally, this metric was called a deficit index, a very generic term. However, the moniker of frailty index was attached to it to explain its relevance to geriatric medicine which deals with frailty, disability, and morbidity. In similar fashion, indices of psychological aging can readily be constructed, for example. The various functional indices simply explore aging at various levels of organization. This reflects a systems approach to aging with its inherent emergent properties.

## Functional Decline and Functional Heterogeneity in Biological Aging

Physical function ability declines with age. This has been assessed variously, but its impact is most obvious when it is examined in terms of activities of daily living (Andersen-Ranberg et al., 1999). Cognitive function also becomes less effective with age (Park and Bischof, 2013). This is not surprising. However, the extent to which function decline varies from individual to individual is less so, and this is apparent for both physical function and cognitive function (McArdle, 2011; Lowsky et al., 2014). At a more basic level, physiologic functions all display gradual failure with age, albeit at different rates of decline (Shock, 1967).

Discrete biomarkers, usually assessed as the level of circulating serum analytes, also show changes with chronological age. In many cases, however, the profiles of change over time are not linear and often display a so-called U-shape (Arbeev et al., 2011). This suggests that at a more granular level functional change with age is complex, and it may reflect the operations of a non-linear system in which interactions between components make their presence known. The important question is how to incorporate this granularity in a meaningful way that does not surrender the ability to interpret the model. One approach to this dilemma uses a multivariate measure of the individual’s divergence from a population centroid that reflects a baseline or normal physiological state at any given age. This multivariate statistic can be interpreted in terms of physiological dysregulation, and it is associated with the transition from a healthy to an unhealthy state (robustness) more so than survival in an unhealthy state (resilience) (Arbeev et al., 2019).

Another approach that utilizes discrete biomarkers assembles them into predictive algorithms (Levine, 2013; Belsky et al., 2015). Often, these algorithms incorporate chronological age. This makes them excellent predictors of mortality (Levine, 2013). This is not surprising, as the Gompertz equation clearly shows that survival decreases exponentially with chronological age (Gavrilov and Gavrilova, 1991). An important application of this approach is its use in predicting functional decrements early in life that are likely to be associated with health later in life, in both cross-sectional and longitudinal modes (Belsky et al., 2015). This has particular value for clinical trials of therapies and interventions that can alter the progress of aging.

DNA methylation marks have also been collected into arrays predictive of chronological age (Hannum et al., 2013; Horvath, 2013). In their various renditions, they are referred to as DNA methylation clocks. The extraordinary coincidence of assigned age using these epigenetic predictors with actual chronological age is unsurprisingly dependent on the heavy conditioning on chronological age of the choice of methylation marks and the predictive algorithm itself. This conditioning is sometimes subtle (Levine et al., 2018). The value of these epigenetic predictors resides in their ability to assign calendar age to samples of unknown origin. They have not been found to predict mortality, or when they do the effect sizes are tiny and only observed with very large samples sizes, usually gathered in meta-analyses (Chen et al., 2016).

The use of predictive algorithms based on biomarkers as applied to young and middle-aged individuals is quite apt. After all, evaluation of the efficacy of a therapeutic or an intervention would reasonably be applied to such individuals before they progress too far along an aging trajectory. Also, we would want to be able to assess the potential outcome early, before late effects on survival are observed many years later. Nevertheless, it is essential to ultimately validate any predictive algorithm according to the “gold standard” in aging research, survival itself. The Gompertz–Makeham equation is the starting point for the derivation of the only universal in aging, to utilize a term from physics (Azbel, 2002). Thus, survival or mortality will always be the “yard stick” in aging research. Certainly, it has propelled many of the major discoveries in the field (Jazwinski, 1996).

The Gompertz equation conceals an inherent conundrum. Despite the exponential increase that the equation depicts, there is always a deviation that it ignores when individual, real-world values are examined. Furthermore, there is a systematic departure from actual mortality rates which becomes apparent around 90 years of age for humans. This departure ultimately becomes a plateau from around 105 years of age on (Barbi et al., 2018).

## Top-Down Modeling of an Aging System

Organisms display complex adaptive behavior, and they interact with the environment. This is the result of their organization, which arises from a multitude of simple, self-assembled local relationships. These function in interactive arrays that are governed by non-linear dynamics. Another way of putting this is that these arrays display emergent properties. Aristotle already recognized this by stating: “The whole is greater than the sum of its parts.” Interactions at a lower level give rise to higher level objects or properties: interactions of molecules form cells, interactions of cells form tissues, interactions of tissues lead to organs, interactions of organs result in the organism, and so on.

Capturing the components at any of the scales of organization is not sufficient to arrive at the behavior at that scale. One must also account for the interactions that make the system complex, non-linear, and hence indeterminate at that scale. This is most readily achieved from a top-down vantage point. Having made this statement, it is important to acknowledge that this must take a top-down, minus-one level approach, because a level is defined by its interacting components.

Deficit indices constitute an uncomplicated way in which to describe the behavior of a complex aging system. Deficit indices have a long history in human aging research and in geriatrics (Rockwood et al., 1999; Mitnitski et al., 2001). They are even being applied to understand rodent aging (Rockwood et al., 2017). A deficit index is constructed from a number of signs, symptoms, marks, and manifestations. The number can be relatively small, about twenty, or much larger, as long as it is statistically sufficient. These deficits should encompass many different body or physiological systems. The deficit index arises by summing the deficits counted and dividing by the total number of deficits assessed. Increasing the number of deficits scored improves deficit index performance (Mitnitski et al., 2017).

Recently, the deficit index has acquired a strong theoretical underpinning. The deficits are represented by the components of a network, in which they can be damaged or undamaged (deficits *per se*) (Rutenberg et al., 2018). By definition, the components are connected by edges. Some of them have more edges than others, performing a more critical role in the network. Damage in this network, whether partial or complete, is propagated across the network or system because of the edges. This rational, systems biology-based nature of the deficit index distinguishes it from other quantitative measures of biological age. In addition, the deficit index is uncomplicated mathematically, as opposed to most of the other measures, and it predicts mortality without the incorporation of chronological age as one of its items.

We have constructed a deficit index we call frailty index-34 (FI_34_), consisting of 34 health and function variables (Kim et al., 2013). The reference to frailty in the name stresses the relevance of the index as a measure of relative health. FI_34_ is a good predictor of mortality (Kim et al., 2013), so it is a measure of biological age as defined earlier. It increases exponentially with calendar age, as we would expect of a predictor of mortality (Kim et al., 2013). Moreover, it distinguishes different patterns of aging, and it is heritable (Kim et al., 2013). FI_34_ also captures the individual variability or heterogeneity of aging among individuals (Kim and Jazwinski, 2015). Although constantly increasing with chronological age across a population, it shows variation among individuals in cross-section and longitudinally.

## Metabolic Dimensions of Biological Age

It is known that energy metabolism slows down with calendar age. We expect that of physical activity energy expenditure, as older individuals are generally less active than younger ones. However, this decline extends to resting metabolic rate (RMR) as well, and thus total energy expenditure is lower in older adults (Kim and Jazwinski, 2015). RMR is ascribed to the energy required to maintain basic body function, and it constitutes 60–70% of total energy expenditure.

The decline in RMR with chronological age that is seen overall manifests itself differently depending on an individual’s biological age (Kim et al., 2014). Counterintuitively, higher FI_34_ in nonagenarians is positively correlated with higher RMR. This is the case in males and females, and this association remains after adjusting for body composition, thyroid hormone levels, insulin-like growth factor 1 (IGF-1), and circulating creatine kinase levels (CK). Thus, there appears to be a metabolic compensation for declining health and the loss of integrated function which the increase in deficits indicates. Damaged components or nodes in the network model of the deficit index are tantamount to loss of edges or connections that measure the functional integration of the system.

The finding that RMR increases with biological age begs the question of the nature of the underlying mechanism(s). Although this relationship occurs in both males and females, these mechanisms differ phenotypically. In males, there is a positive association of CK with FI_34_ in the presence of RMR increase (Kim et al., 2014). This is not observed in females in whom instead there is a negative association of fat-free mass associated with increased FI_34_ and RMR that is not found in males. Thus, in males, tissue damage suggested by increased CK, appears to be involved, while in females it is loss of muscle mass.

Genetic analysis has been applied to access the underlying mechanisms at a more granular level. In males, the CK was associated with regulatory variants in the genes *XRCC6* and *LASS1* (Kim et al., 2016b). *XRRC6* encodes the protein Ku70 which can bind to Bax preventing Bax from binding to mitochondria and initiating an apoptotic cascade that would result in release of creatine kinase from cells thus increasing CK. In females, a different mechanism appears to be involved. Regulatory variants in *UCP2* and *UCP3* are associated with FI_34_, and this association is not found in males (Kim et al., 2016a). These two genes encode mitochondrial inner membrane proteins that are called uncoupling proteins. They function as transporters. The variation in *UCP2* portends a switch in substrate utilized for respiration from glucose to glutamate (Vozza et al., 2014) as FI_34_ increases. There is an interaction between RMR and a *UCP3* variant in the positive association with FI_34_ that indicates an intensification in respiration (Kim et al., 2016a), such that more energy is expended when FI_34_ increases. The relevant variant of *UCP3* is associated with a higher hazard ratio for mortality, which we will come back to later.

## The Gut Microbiota in Biological Aging

Energy metabolism cannot be adequately addressed without consideration of the gut microbiota (Kim and Jazwinski, 2018). These symbiotic denizens of our digestive tract process our diet enhancing its assimilation. They also produce a wide array of signaling molecules. The impact of the gut microbiota goes beyond metabolism to influence inflammation and immunity, leading to age-related degenerative disorders associated with unhealthy aging. The gut microbiota shows relative stability within individuals, but it can vary widely among individuals. The composition of the functional, core gut microbiome is relatively constant in individuals across geographic regions and chronological ages. However, the overall diversity and variability of the gut microbiota increases with calendar age. This increase in the heterogeneity in gut microbiota composition mirrors the increase in individual variation of host physical and cognitive abilities during aging, noted earlier.

Because the relationship of energy metabolism and chronological age is diametrically different from its relationship to biological age, it is important to examine the gut microbiota in the latter context as well. Interestingly, the richness (α-diversity) or intra-individual variation in the gut microbiota declines as a function of biological age (FI_34_), while showing little or no difference with chronological age (Maffei et al., 2017). At the same time, certain bacterial co-abundance networks become apparent with increased biological age, which may be associated with frailty or unhealthy aging. These co-abundance networks may be responsible for the metabolic changes and inflammatory responses that are characteristic of unhealthy aging by disrupting the beneficial interactions of the gut microbiome with host signaling pathways. In other words, the microbiome is an integral part of the network that the deficit index describes. Indeed, the declining richness of the gut microbiota with biological age mirrors the loss of components of this network and the connections they support.

## The Epigenetic Interface Between the Aging Organism and the Environment

The epigenome is often described as the interface between the organism and the environment. The gut microbiota communicates with its host through various signaling pathways and by epigenetic mechanisms (Kim and Jazwinski, 2018). One of these epigenetic mechanisms that has been examined in some detail in the context of biological aging is DNA methylation.

A genome-wide analysis of individual DNA methylation sites and methylated regions in old twins uncovered an association with biological age, as measured using the frailty index, of DNA methylation at CpG sites within the promoter of the *PCDHGA3* gene (Kim et al., 2018). This gene belongs to a cluster of protocadherin genes on chromosome 5. Methylation in this large gene cluster has been associated with age and age-related phenotypes, and it can modulate gene expression. Protocadherins are cell adhesion proteins that appear to also be involved in intracellular signaling. The identification of DNA methylation of protocadherin as a potential player in biological aging is significant because it conjures up the network model of the deficit index. In this case, the protocadherins mediate the interactions between the cells that are the components of the network at this level of organization, by leading to the formation of tissues with their emergent properties.

**FIGURE 1:**
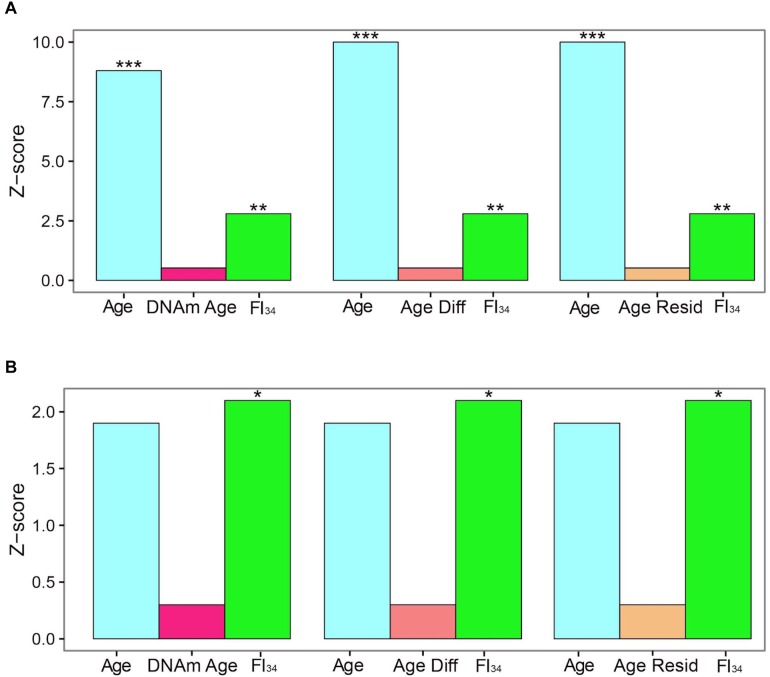
Cox proportional hazards of death. Censored survival data for 262 subjects aged 60–103 from the Louisiana Healthy Aging Study (LHAS) are presented as Z scores. Age, FI_34_, DNA methylation age (DNAm Age), Age Acceleration Difference (Age Diff), and Age Acceleration Residual (Age Resid) are included as covariates in the regressions, as indicated. **(A)** All 262 subjects, **(B)** nonagenarians only (*N* = 161). ^∗^*p* < 0.05; ^∗∗^*p* < 0.01; and ^∗∗∗^*p* < 0.001. This figure has been reproduced from Jazwinski and Kim (2017) under the Creative Commons Attribution 4.0 International License (http://creativecommons.org/licenses/by/4.0/).

The specific DNA methylation of protocadherin described above that is associated with biological age is not the same as the DNA methylation marks selected by their tight association with chronological age, called DNA methylation clocks (see earlier). These DNA methylation clocks perform poorly as predictors of mortality, side by side with age and FI_34_ (Kim et al., 2017). For individuals ≥60 years of age, both chronological age and FI_34_ are significant measures of the hazard of mortality, while thex DNA methylation clock in at least three different versions is not (Figure 1). Interestingly, for individuals ≥90 years of age, only FI_34_ remains a predictor of mortality. A caveat applicable to these tantalizing observations is that they await replication with larger samples in other populations. It is important to note that observed mortality begins to depart from the exponential increase in mortality modeled by the Gompertz equation at age 90 and plateaus at 105 (Barbi et al., 2018). Thus, FI_34_ may account for at least a portion of the actual mortality that is not captured by the Gompertz equation, after the calendar age of 90.

Recently, it has been found that several DNA methylation clocks and composite-biomarker predictors of age are not well correlated with each other (Belsky et al., 2018). The authors concluded that they thus may not measure the same aspects of aging. However, FI_34_ appears superior to the DNA methylation clocks and to chronological age itself at older ages, when they are all compared together. It remains to be seen how FI_34_ performs when assessed together with chronological age and the biomarker algorithms, to determine whether they contribute added information related to the phenotypic variability of biological age.

## Concluding Remarks

**FIGURE 2:**
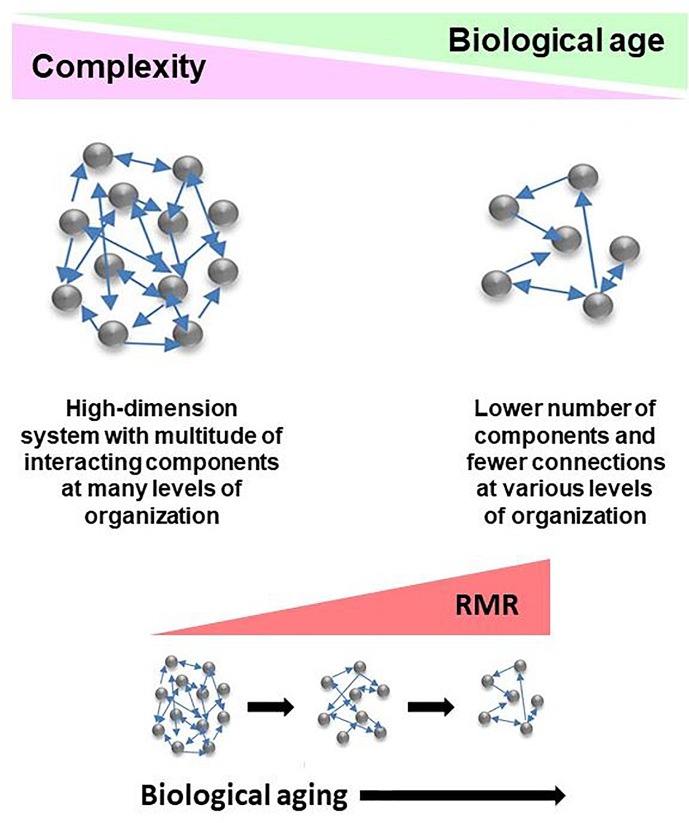
Conceptualization of the deficit/frailty index as a network description of a complex aging system. Complexity declines as a function of biological age. The result is loss of components of networks at various levels of organization, leading to weaker integration due to loss of connections. The aging system consists of the host and its gut microbiome, with the epigenome comprising the interface between them. Losses in integrated function of the aging system pose energetic demands to keep it functioning. This results in the increased resting metabolic rate (RMR) associated with progressing biological age. RMR is the energy expenditure for maintenance of basic body functions. It accounts for 60–70% of total daily energy expenditure.

The network model of the deficit index takes a systems view of biological age, and it explains the complexity of the aging organism by taking into account that the components of the network (system) interact with each other. Damage to these components is what the deficit index quantifies. This damage can be partial or complete. Damaged components are equivalent to weakening or loss of the interactions between them. Thus, damage is propagated throughout the network. Compromised interactions reduce the integration of the system. Thus, complexity decreases as biological age increases (Figure 2), and this is the result of damage and loss of components and the interactions among them. This model extends to all levels of organization, from molecules, to cells, tissues, organs, and organisms. Importantly, the host and the microbiome lose complexity in tandem, with biological age. Thus, the gut microbiota are simply another component of the aging network.

The increase in RMR with biological age or unhealthy aging means, by definition, that more energy is expended for the maintenance of basic body functions. Of course, this may mean that less energy is available for physical activity if total energy expenditure remains constant or decreases, as is the case across the population (Kim and Jazwinski, 2015). The mechanisms underlying the increase in RMR differ in males and females, but in each they involve different aspects of mitochondrial function. The question arises as to how these changes relate to the network described by the deficit index. One possibility is that the increased RMR countervails, at least to some extent, the further loss of complexity of the network. Another alternative is that the heightened RMR is simply the “cost of doing business” as the network loses complexity. We favor the latter interpretation. As mentioned earlier, the variant of *UCP3* that interacts with RMR in association with FI_34_ is also associated with an increase in the hazard ratio of mortality. This suggests that the RMR increase does not favorably impact survival as might be expected if increased energy maintained the status quo.

It will be of interest to determine which of the alternatives mentioned above pertains. In any case, there is little doubt that survival, along with good health outcomes, is supported by a robust network that retains its complexity. It has been found recently (Miller et al., 2018) that individuals with greater functional connectivity in their central executive network displayed better cardiometabolic health than those with lower connectivity. This was the case despite the presence of psychosocial stress, providing a cogent example of resilience. The growth factor BDNF promotes neurogenesis in the brain, and it may enhance resilience in humans (Cahn et al., 2017). BDNF in this case would facilitate the generation of new neurons, perhaps to replace damaged ones, to keep the complexity of the brain neurocircuitry intact.

## Author Contributions

SJ wrote the first draft and made final edits. SK commented on the first draft.

## Conflict of Interest Statement

The authors declare that the research was conducted in the absence of any commercial or financial relationships that could be construed as a potential conflict of interest.
